# Parental Perceptions on the Importance of Nutrients for Children with Autism Spectrum Disorder (ASD) and the Coping Strategies: A Qualitative Study

**DOI:** 10.3390/nu15071608

**Published:** 2023-03-26

**Authors:** Woan Yin Tan, Nur Hana Hamzaid, Norhayati Ibrahim

**Affiliations:** 1 Dietetic Program, Faculty of Health Sciences, Universiti Kebangsaan Malaysia, Kuala Lumpur 50300, Malaysia; 2 Center of Rehabilitation and Special Needs Studies (iCaRehab), Faculty of Health Sciences, Universiti Kebangsaan Malaysia, Kuala Lumpur 50300, Malaysia; 3 Center of Aging and Wellness (H-Care), Faculty of Health Sciences, Universiti Kebangsaan Malaysia, Kuala Lumpur 50300, Malaysia

**Keywords:** autism spectrum disorder, children, parents, perceptions, nutrients, coping strategies, semi-structured interview, community-rehabilitation program center

## Abstract

Autism Spectrum Disorder (ASD) is a developmental disorder that comes with co-occurring eating behavior such as limited food varieties, selective food intake, and repetitive eating patterns, contributing to significant challenges for their parents. Many parents find mealtimes difficult and develop anxiety or stress about their child’s nutrition, health, and overall well-being. Hence, parents must equip themselves with the correct awareness and nutrition knowledge. An online open-ended semi-structured interview was conducted among fifteen parents from the community-rehabilitation program center to explore their understanding of nutrition’s importance and the possible coping strategies when facing challenges. The interview sessions were recorded, followed by three researchers’ coding processes. Data were then subjected to thematic analysis. The interview sessions suggested that the parents were aware of the autism trait eating behavior and had a general knowledge about nutrition. However, it was quite challenging when it came to preparation. Nevertheless, the parents are able to manage the challenges with unique kinds of coping strategies. In addition, a complete educational dietary intervention program including psychosocial aspects for parents is recommended for better effectiveness.

## 1. Introduction

Autism Spectrum Disorder (ASD) is a developmental disorder that can lead to challenges in children’s social skills, communication, and behavior [1]. Children with ASD usually show social and communication problems such as an inability to perform in everyday conversation, lack of interest, and finding it hard to respond to social signs such as eye contact, facial expressions, or making friends [2]. At present, globally, the most extensive surveillance for the prevalence of ASD is carried out by the ADMM (Autism and Developmental Disabilities Monitoring) Network established by the Centers for Disease Control and Prevention (CDC) [3,4]. A recent report in 2016, 1 in 54 children are diagnosed with ASD, with boys four times higher than girls, and surprisingly, this figure has increased by 2.5 times since the year 2000 (1 in 150 children) [4]. In Malaysia, there is no local data available for the prevalence of autism [5]. However, a smaller-scale study by the Ministry of Health (MOH) with the application of the Modified Checklist for Autism in Toddlers (M-Chat) between children of ages ranging from 18 to 26 months shows a rate of 1.6 in 1000 are diagnosed with ASD [5].

Part of the clinical symptoms of ASD involves atypical eating behavior. Although eating behavior such as food refusal is also observed in the general pediatric population, it is surprising to know that the percentage of food refusal in children with ASD is significantly higher, with the rates ranging from 51% to 89% [6]. This indirectly contributes to part of the parental stress [7]. However, the specific cause for food refusal is still poorly understood [7]. From these previous studies, the authors point out that atypical eating behavior is observed since infancy during breastfeeding such as difficulties in sucking [8], vigorous [9], and or frequent stops during breastfeeding [10]. As the child progresses for weaning, difficulties in the food texture transformation, from pureed texture to solid, are also observed by their mothers [11]. The children take more time in accepting solid food texture [12] and also have a slower progression in using a cup and fork [13].

Once entering childhood or adolescence, the most common problematic eating behavior is food selectivity [14,15,16,17]. Children with ASD may reject food for various underlying physiological and or behavioral reasons [18]. Parents of children with ASD identified food texture, appearance, brand, packaging, temperature, food presentation, color, taste, and aroma as factors that affect their child’s preferences [19,20,21]. Food selectivity is also linked with nutrient deficiencies [22,23], probably due to the lack of food varieties [23], favoring energy-dense food [24], insufficient intake of fruits and vegetables, and high intake of sugary beverages [25]. All these factors suggest that children with ASD will encounter an undesirable nutritional status, either underweight, overweight, and/or obese. Recent findings from a cross-sectional study in Kuala Lumpur exploring the parents’ and special educators’ perceptions of healthy eating found that the interviewed participants had limited understanding regarding a balanced dietary intake and nutritional requirements for the children [26]. Likewise, another recent finding from a local cross-sectional study observed a high prevalence rate of overweight (21.9%) and obesity (11.3%), with 91.4% of the participants having eating behavior problems related to food selectivity [27].

Family involvement, specifically the parents, is one of the most important factors for children with Autism Spectrum Disorder (ASD) development [28]. It is reported that early interventions for young children with ASD can yield positive developments in their cognitive and communication skills and improve their ASD symptoms [29,30,31]. Parenting a young child with ASD can be extremely challenging and have both positive and negative features, ranging from greater resource needs to higher levels of parenting-related stress and positive personal development for family members. It is also proposed that parental satisfaction and the child impacts are influenced by each other in a transactional manner [32]. The participation of parents might be able to improve the parents’ well-being which includes improving the positive feeling [33,34], helping in reducing stress [35], and also parents’ self-confidence [36]. When compared to parents of typically developing children [37,38], parents of children with Down Syndrome [39], and parents of children with other disabilities [40]; parents of children with ASD generally report higher levels of parenting stress [37]. Coping strategies can be one of the mechanisms for the parents of children with ASD to adapt to the stress [41]; additionally, with ASD, more severe and long-lasting core features can bring significant impacts on family functioning [42,43,44,45]. Coping is defined as “constantly changing cognitive, emotional and behavioral efforts to manage specific external and/or internal demands that are challenging or exceeding the person’s resources” [46].

Considering that parents are one of the key features in their child’s involvement in all kinds of intervention programs, it is vital to assess parents’ perceptions of their child’s eating behavior and the level of understanding of healthy eating, mainly to prevent the occurrence of underweight, overweight or obesity. Previous quantitative studies conducted in Malaysia suggested Malaysian parents of children with ASD have limited knowledge relating to healthy eating. Instead, they prefer to use nutritional supplements to improve their child’s nutritional status [26,47,48,49]. However, the literature is limited to parents’ perception of the impact of ASD on their child’s nutritional status and their coping mechanisms. Therefore, the objective of the present study is to explore the parental perception of their children’s eating behavior, the parents’ understanding of healthy eating, and also their coping strategies toward the challenges met to enhance a better nutrition outcome.

## 2. Materials and Methods

### 2.1. Study Design and Study Sample

Online open-ended, semi-structured telephone interviews with parents of children with ASD were conducted in this qualitative cross-sectional study. This study was approved and permitted by the Research and Ethics Committee of Universiti Kebangsaan Malaysia (JEP-2020-713) and the Department of Social Welfare (JKM) Malaysia (JKMM 100/12/5/2:2021/034). The parents were also provided consent to participate in this qualitative study. This present study was conducted in one of the states in Malaysia—Melaka. The study subjects were selected from the government-based CBR center (Community-Rehabilitation Program) and a non-government organization NASOM (National Autism Society of Malaysia) in Melaka.

CBR is a program constructed by the Department of Disabled Development (JPPWD) by JKM Malaysia. It serves as a part of the local community’s development strategies in terms of rehabilitation, training, education, equalization in opportunities, and social integration for individuals with disabilities. An individual with disabilities diagnosed by the medical officer and registered with the social welfare department was eligible to join the program. Likewise, for children with ASD, as early as two years old, the child can register under the program.

Participants in the present study fulfill the inclusion criteria of having a child with ASD aged between 3 and 11 years old. It was conducted individually with the parents. The parents were either the father or mother of the children with ASD attending the CBR or NASOM center. This interview was facilitated by a structured question guide (Table 1) that focused on these three topics: parental perceptions of the child’s eating behavior, parental perceptions of healthy eating, and parental coping strategies. In addition to that, a self-administered questionnaire was given to the parents to fill in regarding demographic information to obtain information about their children’s age, gender, ethnicity, and the parent’s education level, occupation, and total household income.

### 2.2. Data Analysis

The study team was comprised of three members. All interviews were conducted by T.W.Y. who is the main principal investigator. All interviews were recorded digitally and transcribed verbatim into a word document and analyzed individually by the study team members. The recorded audio was first transcribed, followed by a coding process by the researchers. The coding process was conducted by three researchers (T.W.Y., N.H.H., N.I.). To obtain important patterns in the data, thematic analysis was conducted using Braun and Clarke’s 2006 six-phase framework. This process involves finding patterns within the data by reading and re-reading the transcription (T.W.Y.), creating preliminary codes that are pertinent to interesting features in the data (T.W.Y.), looking for themes (T.W.Y., N.H.H., N.I.)), checking to see if those themes related back to the preliminary codes (T.W.Y.), identify the themes and group the themes (T.W.Y., N.H.H., N.I.). This process is repeated over multiple sessions. The following example in Table 2 gives an overview of the process.

## 3. Results

### 3.1. Participant Description

There was a total of 15 participants in this study group according to the data saturation. All children who participated in the study were in an age range from 5 to 11 years old with the majority of children with ASD being boys (73%). Parents generally completed their studies until degree level (67%) with the lowest at primary school education. The majority of the participants were housewives (47%), followed by public workers (33%), and self-employed (20%). All of these participants were a mixture of low and middle-income groups. This is shown in Table 3, demographics of participants. 

### 3.2. Themes

**Figure 1:**
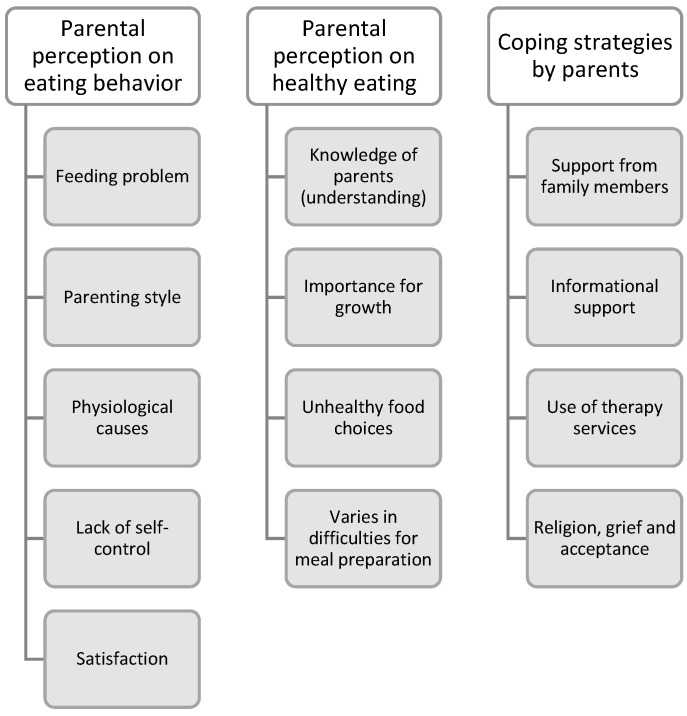
The summary of emerging themes.

An average of four to five primary themes were identified, along with the exploration topics (Figure 1). The themes that are identified are supported by related quotes from this interview session. The quotes were taken directly from the transcripts; hence, there will be no grammatical corrections to edit. Participants are labeled as “P”.

#### 3.2.1. Parental Perception of Eating Behavior

Theme 1: Feeding problem

The interview started by asking the parents to describe their child’s eating behavior. Ten of fifteen parents said that they experience feeding problems with their children during eating time. The feeding problem that was mentioned by the majority of parents was the child’s selective behavior toward specific textures, smells, colors, and food choices, as illustrated, respectively, by the following quotes.

“Oh okay…my child, he seems like a picky eater. He doesn’t like soft mushy texture foods. He likes crunchy food. He refuses soft texture. [The mom describes his son’s eating behavior] And his eating behavior is seasonal. He used to take rice previously. But once he feels bored over it, he will refuse to eat. He will then choose others food like bread. Then the cycle repeats, when he feels bored over certain types of food; then, he will choose other types of food again.” (P8)

The same parent also added his child’s food refusal for soft textures related to fruit intake.

“Don’t mention fruits. He refuses cause of the texture of the fruit. He is only keen to try it by biting once or licking with his tongue, then that’s it. Even like vegetables, I have become crispy texture; only, then is he willing to eat.” (P8)

Other remarks by the parents,

“…my child always with spaghetti. He always eats spaghetti. Sometimes will eat rice; but only with his favorite dish. He likes fries, fried prawn crackers. My child refuses new food.” (P11)

“I notice that my child is somehow like a picky eater; because he chooses his food. When we offer him others, he rejects the food. My child will eat that same type of food daily, keep repeating, and won’t feel bored.” (P12)

“My child is picky with his food. He doesn’t like something too salty or too soft in texture. He only likes to eat cocoa-based cereals. Or he only takes “nasi lemak”, that’s all. He doesn’t like fruits and vegetables. Even if we hide the vegetables by blending them, he seems to know, then he refuses to eat.” (P13)

Another parent commented that their child has a rigid routine of food intake.

“Yes! [The parent agrees with the son eating behavior] He is rigid with his routine; and his food choices. If he insists on that food, [hah—parent expression], that’s the only food he chooses. For example, for breakfast, he only wants fried vermicelli. Then followed by white rice and fried chicken, then white rice with egg…and it must be scrambled egg. These are the only food groups he takes. Yea [Agree from a parent], his eating behavior is so rigid.” (P10)

Interestingly, a parent mentioned the selectivity of the child regarding specific eating places and utensils used.

“Yes, sometimes, he will only focus on eating during his eating time. Do not disturb him. And then, if, let’s say, he already chose a destined place to eat, he will not change to another place anymore; that is the only place he likes. He will wait for his siblings to finish using that place or chair. And then even the plate, he also got his specific plate colors to use, like pink or dark purple. He will only go for that specific place and plate.” (P9)

Last but not least, one of the parents raised their concern about the child’s poor gross motor skills, which delayed the child’s developmental progression.

“Yup, I need to feed my child. But for foods like fish crackers or fries, he still can eat by himself. But he can’t hold properly; he is not strong yet.”

Theme 2: Parenting styles

Other than feeding problems observed from the autistic child itself, different parenting styles were also noticed through the interviews that were not aware by the parents themselves.

“…previously my child refused to eat. This is based on my opinion. I’m not too sure about the other parents. But in my opinion, I will act in this way. I will not follow their demand. Some parents sympathize with their children and follow their demands, but not me. When my child refuses to accept new foods, I will explain to them. You need it to keep telling, and they will know the food is tasty after they try it.” (P5)

Another remark by the parent was,

“We must guide him; we cannot just leave him like that. They should eat the same as us. But when I sent him to an early intervention program, I noticed that most of them liked to eat chicken. So, I did was, I told the principal in charge to give him the foods that they serve. If he doesn’t want to eat, then you just monitor him. I didn’t pack him any food; I just packed him mineral water. The results are okay; he can accept little by little.” (P9)

Nevertheless, the parents keep modifying the food texture so that their child is willing to accept new foods and it can help to include more varieties in their daily menu.

“…I know my child doesn’t like fruits and vegetables. But, no matter how he needs to take fruits and vegetables. So, I will dip them with flour and egg for vegetables to make them crispy and fry them. Thank God, he eats it.” (P8)

“…For now, for your information, I process food like nuggets and sausages for him from scratch. Because he likes to eat fast food, it will not be a wise choice if we keep providing him with it. So, I will process the food myself and add in vegetables inside.” (P12)

Regardless of the family’s socioeconomic status, the parents always regard the child’s mealtime as the main priority.

“Eating is essential…no matter where we go, we need to eat. But for my child, because he is a special child, so even though we do not have much money, as a parent, we still need to prioritize him [for buying food]. He is not like us, we can be flexible, but he can’t. So, we have to fulfill the foods that he wants.” (P12)

Surprisingly, overprotection is also seen in one of the parents toward her children.

“For now, I’m feeding all of my children. I refuse to let them eat by themselves as they will not finish the food and mess up the places. Hence, every time, I will feed them, all of them.” (P4)

When further probing the same parent on whether the child is able to eat by themselves, she said,

“…he can eat himself, but I won’t allow it. This includes my eldest son (a typical development child); I will also feed him. For example, I’m serving them fish, but I’m afraid they aren’t aware of the fish bones. And one more thing, they will be picky if they eat by themselves. So, if I’m the one that feeds them, everything that I feed, they will eat it.” (P4)

Theme 3: Physiological causes

Three out of fifteen parents have reported their children’s underlying physiological effects from the autism spectrum disorder traits that significantly affect the child during mealtime. This causes mealtime difficulties for the parents and requires an alternative method to ease them. These parents reported that their child could not stay focused, had no feeling of hunger, and showed no interest in food at all.

“He…during his mealtime, he is unable to sit quietly. So, I must let him watch something [gadget/TV] for a while. Yup, watch television or let him watch the video through his handphone to focus during mealtime. And I still feed him because he is not good at self-feeding.” (P2)

“We feel very challenged when feeding my child because his upper limbs will keep moving uncontrollably. We only can feed him when he knows how to open his mouth, and we need to trigger him to open his mouth. He seems like he doesn’t have any hunger feeling.” (P11)

“My child...he can’t sit, and he doesn’t want to eat. He even refuses to look into the food…he has minimal interest in the food.” (P14)

Theme 4: Lack of self-control

Two out of fifteen interviewed parents reported that their child lacks self-control regarding satiety. Both of these parents face the same issue where their child will frequently eat between meals, so, they have to provide food for them constantly. Parents think that it is an unhealthy habit for the child.

A parent mentioned this with an example,

“I don’t know how, but my child will keep repeating, asking for food. He is still non-verbal. But that doesn’t stop him from requesting food. [laughing] He will bring you to the kitchen because he wants to eat. To ease our convenience, we will prepare light snacks like fries. For example, at noon, I gave him fries, he would finish them (one bowl of it), then he would play himself. But after that, he will ask for food again…is like every 30mins he will ask again…” (P12)

Another parent also said the same,

“For her…if the food that she likes…she will eat a lot until I need to hide it. I usually won’t give her too much because I know she doesn’t know how to stop. [Laugh] I think she doesn’t know the feeling of fullness. Like us, when we know we are full, we will stop eating. But for her, for example, yesterday, I provided her with fried noodles [I control the portion]. But after half an hour, she requested cream crackers and ate around ten pieces. I even purposely stop buying it because she will eat a lot and be unable to control it.” (P14)

Theme 5: Satisfaction

As commonly reported, children with autism spectrum disorder are usually associated with feeding problems such as picky eating or food refusal. This was not an exception in this interview, as shown in the previous themes. However, on the other hand, it is interesting to find out that four out of fifteen parents reported that they have no problems with their child’s eating behavior. They have no difficulties in preparing and serving food for their child.

“If is my child, I don’t think there is a problem with his eating behavior. He is okay so far. He can eat all. If he wants to talk about his eating behavior, I would only say it is hard to sit while eating. But he will eat all; he will finish all the food.” (P1)

“He is not choosy on his foods. He likes to eat. Everything you give it to him (rice, fruits, spicy, sweet, sour, etc.), he will finish all.” (P1)

Another parent also states that they disagree with the general fact that autistic children are picky eaters, as illustrated by the following quote.

“Okay…got some autistic children. They are like a picky eater. But I don’t think this happens to my child. It is easy to handle my child during mealtime. She eats every food group–chicken, fish, and vegetables, she eats. For instance, some autistic children are sensitive to the aroma, but this is not happening to my child. So, to me, it is not the same. My child can eat all.” (P2)

#### 3.2.2. Parental Perception of Healthy Eating

Theme 1 Knowledge or understanding of parents about healthy eating

All parents interviewed in this study generally had a common basic understanding or knowledge about healthy eating when being asked. The parents understand that healthy eating should come from specific food groups or should be a complete meal and give a satiety feeling, as illustrated by the following quotes.

“From my understanding, healthy eating to me is about vegetables, fruits, and rice. That’s all on what I understand, as simple as this [laughing], and, must be able to feel full. Junk food is not healthy eating.” (P1)

“Healthy eating is like must have fish, chicken, vegetables. Have to be balanced, the foods need to be balanced. Yes, balance diet. It is not about sweet food.” (P2)

“Okay, healthy eating is like balanced eating, that is what I can understand. Have to make sure his nutrition is enough, needs to have protein, carbohydrate, and no sugary things.” (P3)

One parent said,

“I hope that I can help him with food varieties since he is a picky eater; I am worried if his diet lacks fiber or any other nutrients. If possible, I hope he can have varieties of food in terms of the sources.” (P8)

Nevertheless, one parent was concerned about their child’s eating intake and thought supplementation would be a healthier choice.

“Supposing consists of protein, consists of basic meals. But because he dislikes certain food, sometimes I will provide him with multivitamins. And now you look at his weight increasing, so I think better I should stop the vitamin [laughing].” (P9)

Meanwhile, only one parent refers to the Malaysian food pyramid as healthy eating guidelines.

“Okay, healthy eating is like referring back to our food pyramid. Means, we have to provide the quantity that the food pyramid had suggested, including the vegetables, fish, and rice.” (P12)

Theme 2 Important for growth

Three out of fifteen parents also see healthy eating as part of the requirements for their children to have normal physical development.

“Healthy eating is important for his growth.” (P2)

“Healthy eating is very important, important for his growth. Even though he is a special child, he still needs to grow like usual kids. Am I correct? Autism affects his behavior, but he is growing still the same.” (P3)

“They have to eat healthily. It is essential as it will affect their development in general and also their brain function.” (P4)

Theme 3 Unhealthy food choices

Nevertheless, there are also voices from parents mentioning sugary intake. The parents think that sugary intake is one of the unhealthy choices for their kids. This can be further explained by the effect observed on the child.

“…we know about our child; he, is not suitable with too much intake of sugary food, he will become hyperactive and starts giggling by himself and also running around. Then we ended up feeling tired as parent, so we have to control his intake of sugar.” (P3)

“Healthy eating is important, especially for his growth. If able to eat healthily, I think it can help him behave well. It will affect his emotion, especially towards sweetened food or beverages, because it affects his sleep cycle, from my observation.” (P8)

Other than sugary intake, one of the parents also raised her concern about the food quality and said,

“Okay, I only buy free-range chicken; the same goes for the egg. I am worried and anxious about the quality of the food. And if it is vegetables, usually I’ll buy red spinach. Nowadays, I get so much news about animal injection or polluted plantations that I won’t buy it. Their health is my main priority now.”

Theme 4 Varieties in difficulties for healthy eating meal preparation

When it comes to healthy eating meal preparation for autistic children, parents commonly experience challenges due to the autistic child’s eating behavior. Two different phenomena can be observed in this study. Half of the fifteen parents do not experience any difficulties in healthy meal preparation, but the other half feel the challenges. This can be further explained by parents who do not have no problems or issues with their child’s eating behavior.

“I think I’m quite okay with it because my child is not a picky eater, so it is quite easy for me to prepare the food. It is not the same as mentioned, where I have to prepare differently for him. To me, it is easy, is easy for me to cook for everyone to eat.” (P1)

“I feel it is easy for me to prepare healthy food for my child. I didn’t help him much; I’ll just prepare fruits and vegetables for him [extra]. So, when he is hungry, he will take himself. I feel there are not many issues for him.” (P3)

As opposed to this, one parent felt that the child’s eating behavior added to the family’s poor socioeconomic status and increased the difficulties level in healthy meal preparation for the child.

“Challenges arise when the food (food availability) that we prepare, my child refuses. So, we have to find him the alternatives that my child will accept (with our affordability).” (P12)

In addition to this, another parent reflected that it is harder to help their child with healthy eating preparation when the child is involved in the cooking preparation and decides the menu of the meal.

“It is difficult. Firstly, the portion size of the food is already more than the recommended; second, the unhealthy food choices that my child likes. She likes sweetened food. For example, she already had her breakfast, but I noticed she would go and make herself a cup of hot cocoa-sweetened drink after a while. She knows where I usually hide it.” (P14)

As well as another remark from the parent is that they are afraid of triggering their child,

“I feel there is difficulty to help my child with healthy eating—sometimes we do not have much encouragement to control his portion intake because he will misbehave (scream) if we do not go according to his request.” (P15)

#### 3.2.3. Coping Strategies

Children with special needs always require more attention than typically developing children. This can arise from underlying factors such as a medical condition that requires further medical treatment or disabilities from physical development that cannot carry out their daily living tasks. Eventually, the parents were forced to deal with unforeseen demands, leading to different forms of tension—the same goes for these fifteen participating parents. It was notable that these fifteen parents faced some tension when their children misbehaved. However, interestingly, these parents claimed that they could cope with it.

Theme 1 Support from family members

This starts with a parent mentioning that support from family members and their spouse makes a significant impact on coping.

“Oh, to be honest, it is quite hard to answer, but it is okay; I can…[laughing] I do not deny; it is stressful. Furthermore, with the current situation [movement control order], we can’t go out quite often. But I feel grateful because my child does not show tantrums. He is not like other autistic children; [strongly affirming] he is not. And, I feel less stressed because he got his elder sister that will help me keep an eye on him, and I am so lucky to have my parents [children’s grandparents] that stay near me; so that I can bring him to their house. My husband helps me a lot too. When he is not working, he will take care of him. We did set up a rule where we will take turns to clean up our son if he passes motion [laughing].” (P1)

“…and one more thing, my mom supports me the most. She keeps encouraging me; she is my pillar of support. Sometimes she will stay over at my house and help me take care of my child. So, when you know someone is supporting you, eventually, you won’t feel stressed. I am grateful for everything.” (P3)

“...and supports from family members. Even though my husband is very busy, he always is my listener and gives opinions.” (P8)

Theme 2 Informational support

Furthermore, with the rise in convenience and flexibility of social media, such information can be accessed primarily by support groups from the same community. The same parents also added,

“...social media…I joined the autism social media group page; and learned a lot about my child’s character, how to handle my child, and the suitable toys for an autistic child because they are not like the typical child. When you buy them toys, they will give you an excited reaction. Autism children will not give you this kind of response. So, for my child, I plan to train him to play with puzzles to help with his focus and eye contact. I did a lot of readings from the autism club social media page and also my own research.” (P3)

“Of course, it is stressful, but I will do lots of reading related to my autistic child; and how I can handle them well. Besides reading, I will also join online classes related to autism; it is like doing your research to apply it to my child. The stress is not daily, maybe once in 2 to 3 weeks. The stress is not affecting my daily routine.” (P8)

Theme 3 Use of therapy services

Other than that, parents with more money will send their children to paid therapy services to help solve the problem.

“To me, so far, my child is okay. When he was small (a baby), he was easy to take care of. But when he was around 5 to 6 years old, he started to develop tantrums and face sleep disturbance. So, we went for paid therapy services to learn about handling his sleep disturbance, like brushing, and covering him up to calm him. We are able to see the effects on him; he is getting better.” (P2)

“It is to this extent…when he eats curry puff, he only eats the crust and removes the inner ingredients of it. Hence, I need to send him to paid therapy services just to train him on eating curry puff.” (P14)

Theme 4 Religion, grief, and acceptance

Nevertheless, some parents mentioned that they can see their progression, in terms of emotion and physical handling, from finding out that their child was diagnosed with autism until now. Parents evolved from denial to acceptance and belief in their religion.

“Previously, I do feel stressed…the moment when I know my child is autistic. I couldn’t accept it at that moment; even my husband also can’t accept it. Because the way my child acts to us is like he is a normal child. But, after a few months, I am getting better with it; I can accept it. I keep doing self-talk [laughing]. I can slowly accept it. And I always told myself that some people even a tougher life than us. We are way better than them, and I think this is what God wants us to experience, so yeah, we accept that our child is an autistic child.” (P9)

“…In my first and second years of knowing my child is autistic, I cry badly. Especially when others criticize your child, I can’t handle it. But right now, I’m getting better. I will slowly understand my child and find out the solution for it. So, the tension will be lesser, and you will be able to handle it better.” (P12)

“When she was small, the stress level was high. Because she is still small and unable to understand our instructions. The most challenging part was she didn’t want to sit down quietly. We couldn’t go anywhere, and my social life had changed. But, as she grows up, we can slowly adapt it in our life, so we are getting better now. And right now, she’s able to eat by herself; she can follow instructions. So, whenever I feel tension, I will say this to myself—even though she is autistic, we have to feel gratitude that our child can be independent in her basic life. And one more thing, pray to God. Don’t compare your own with others. We won’t know the hardship of others.” (P14)

## 4. Discussion

Narrative findings from this semi-structured qualitative study suggest five core themes for the parental perception of eating behavior, four core themes for parental perception of healthy eating, and four core themes for parental coping strategies. All of these core themes, when looking into a whole, suggest that, regardless of whether the parents have a precise understanding of healthy eating, regardless of the family’s socioeconomic status, all of them reflected that it is fundamental to ensure that their child can eat healthily through multiple efforts. It is agreed by the parents that they do feel challenged when preparing meals for their child due to their food selectivity behavior; yet, this group of parents also showed their unmeasurable efforts in helping their child to mold better eating behaviors, hoping to achieve higher nutrient intake. Taking care of autistic children requires much more attention than a typical development child, and this study also reflects that the parents experience stress. However, the themes also suggest the parents’ ability to cope and adapt. The coping and adaptation levels suggested increased breakthroughs as the child grows. Furthermore, an exciting theme also arises from this study group where some parents are satisfied with their child’s eating behavior, with just mild unsatisfactory feelings about their social and communication impairment. All of these would deliver refreshing inputs to researchers, educational practice, and the clinical practitioner.

### 4.1. Parental Perception of Eating Behavior

At most, children with autism spectrum disorder have always been categorized as picky eaters due to their food selectivity behavior; in addition, given their nutritional status, regardless of body weight or dietary adequacy, they have also always been focused on by healthcare professionals. The feeding problem is the most common topic among parents regarding mealtimes. Beyond the behavioral, social, and communication difficulties, co-morbid feeding problems are a considerable challenge for parents and providers since autism spectrum disorder is linked with a wide range of feeding issues [50]. Undeniably, the prevalence rate of underweight or overweight and obese children with autism spectrum disorder is higher than the typically developing children of the same age; and it was said to be due to autistic children’s food selectivity behavior [12].

A previous study has proposed that children with autism spectrum disorder exhibit a variety of feeding challenges, with the frequent need for extra support during mealtimes [51]. The current study is not exceptional. For example, the current study reported that one of the significant findings for the child feeding problem is their food selectivity towards texture, aroma, color, taste, and food choices, which can also be categorized as food refusal or described as a picky eater. Moreover, it has been proposed that this kind of food selectivity behavior based on the characteristics of food might be due to the disability in sensory processing and oral or tactile sensitivity [7]. Eating involves receiving information concurrently from various senses, including vision, touch, taste, and smell. Hence, children struggling with sensory processing might show more rejection of the foods [52]. Other than characteristics of the food, dislike of foods being mixed might also relate to sensory sensitivity, or it could also be due to the need for sameness, such as only preferring to have only one kind of food presenting at that moment only [7]. Moreover, for food selectivity according to color, evidence shows less relation with sensory processing [7]. Above all, these occurrences were similar to our current study. Gross motor skill is also another factor that might affect the child-eating behavior—for instance, being unable to pick up food and perform self-feed practices. In general, around 87% of autistic children experience motor difficulty [53]. To date, reports suggested that the autistic population was at a higher rate of gross and fine motor skills deficits. This includes an inability of essential motor control, the inability to perform skilled motor gestures, and irregular motor learning patterns, which disrupts the autistic population’s ability to reach and grasp an object [54]. Moreover, the main effect of the motor impairments can be seen in their daily living activity with tasks as easy as grabbing a spoon [55].

Parents concerned with their child’s development will eventually pay attention to their child’s eating intake. Moreover, when dealing with a special needs child, parents will eventually feel insecure when they cannot deliver the meals for their children. Hence, parents will do anything if the food reaches the child’s mouth. The current study suggests that parents will use gadgets for children with short attention spans during mealtimes and/or have minimal interest in food, which is similar to the two previous studies [56,57]. However, this is without knowing that exposing children to social media during mealtime is one of the forms of pressuring the child to eat. Moreover, to our knowledge, pressuring the child to eat will indirectly create negative messages for the child [58]. Such negative reflections are the possibility of the child having less liking for the food, and less willingness to eat the food, and the child might not learn that eating is a necessary routine in daily life [58]. Apart from that, it is said that overeating or being overweight could come from pressure [59]. Other than that, there is also a finding from this study stating that the child cannot perform self-control when eating and this raises concern from a few parents. The parents think that the child has no satiety cues. It has been proposed in a few studies stating the ineffective processing of their brain signaling (anterior insula—AI) in sending messages to their body [60,61]. This AI helps signal the brain to respond to salient stimuli that include thirst, hunger, temperature, satiety, heartbeat, pain, itch, touch, gustation, vasomotor activity, and muscular and visceral sensations [62]. Therefore, this could also be the possible explanation for the lack of satiety cues.

While this study supports the finding in similar ways, some parents disagree with the feeding problem in children with autism. In contrast, parents reported no problem with their children’s eating behavior. To date, there are no studies that mention the satisfaction of parents with the eating behavior of autistic children. The possible explanation for such findings in this study is that these few children might be from the mild stage of autism spectrum disorder. A common characteristic among these few children reported is that they do not develop any difficulties in food transition since they are small. Parents of these children also do not find any difficulties in preparing food.

As mentioned previously, one of the exciting parts of this finding was the different parenting styles that the participants showed. Parenting and feeding styles have a significant impact on a child’s weight and food consumption. Nonetheless, the studies are still not strong among the autism spectrum disorder population [50]. In general, parent strategies that help increase food acceptability in children consist of preparing a wide range of meals available in the household, consistently introducing the child to new and similar foods, providing positive encouragement, and parental modeling [63]. Yet, eating practices such as controlling child intake, pressuring the child to eat, limiting their intake, or using the reward approach negatively affect their mealtime behavior. It was proposed that parents are more easily affected emotionally when handling children’s mealtimes and might change how parents engage with them [64]. For instance, it was reported that children with parents of authoritarian feeding styles would develop a less favored intake of fruits, vegetables, and juices, whereas children with parents of authoritative feeding styles reflected greater choices of fruits and vegetables, and lesser junk food [65,66,67]. In addition, a recent study further supports the relationships between parenting style and parenting feeding strategies with the child’s picky eating behavior [68]. This study found that parenting style does link with the strategies they use to help their children during mealtime and reduce the rate of exhibiting picky eating characteristics [68]. It was concluded that changes were noticed in children’s food acceptance with different parenting styles. A higher rate of picky eating was also noticed with parents who use authoritarian and permissive characteristics such as limiting, rewarding, and disapproving during mealtime [68]. In opposition, when authoritative practices such as praising, modeling, and involving children, it was noticed that the child exhibited a lower rate of picky behaviors [68].

The current study did not assess parenting style. However, this theme emerges unexpectedly when parents put effort into helping their children during mealtime, especially when it comes to their picky eating characteristics. Parents or other family members will spend ample time with the child to guide, encourage, and mold them into better mealtime behaviors. Yet, some parents use rewards to limit the child’s in exploring themselves during mealtime. This group of studies shows a different level of picky eating with the different parenting efforts shown. Still, it is inconclusive since no measurement tool is applied in this study to measure the parenting style. To our knowledge, a limited study looks into the association between general parenting style and picky eating behaviors in autistic children. Nonetheless, a study examines the association between parenting style and autistic children’s body weight status. This previous study found no relationship between parenting style and the child’s weight status, regardless of authoritative, authoritarian, or permissive parenting style [45].

### 4.2. Parental Perceptions of Healthy Eating

Another topic explored in this study is the perception of parents toward healthy eating. Concern for an autistic child can be challenging in many aspects, and healthy eating is no exception. It is hard for children with autism spectrum disorder to achieve nutritious and balanced meals. Hence, this increases the risk of certain nutrient deficiencies. Therefore, it is essential to find out what parents think about healthy eating. Various understandings of healthy eating are observed in this present study group.

Parents support character in the concept of healthy eating; however, the definition of healthy maybe not be uniformly held. Moreover, parents’ strategies to promote healthy eating may differ. For instance, some parents believe that children must obtain permission to snack. In contrast, some parents believe that allowing children to consume snacks without permission can indirectly prevent them from being obsessive about it. In autism, the majority of the caretakers will seek alternative methods or non-medical treatment approaches, hoping to improve their symptoms [69]. The most common dietary interventions that applied were gluten and casein-free diets, which means eliminating gluten and casein from the child’s diet, and using nutritional supplements such as omega-3s, vitamins, minerals, amino acids, and herbs [70]. However, according to studies, the effectiveness of these dietary interventions is still debatable [71,72]. The elimination of gluten and casein is not recommended as it can be costly and has a limited range of varieties. Furthermore, it is hard to access in a Malaysian setting. The limited range of varieties may also lead to certain nutrient deficiencies.

From this study group, only one parent suggested the link between healthy eating and the food pyramid in Malaysia. As stated, the Malaysian food pyramid can be found in the first key message of the Malaysia Dietary Guideline (MDG)— “eat a variety of foods within your recommended intake.” It emphasizes the recommended food groups and serving sizes that the public can adopt. MDG is a collection of the latest evidence-science-based nutrition and recommended physical activity made by the technical working group. MDG aims to deliver suggestions for developing healthy eating theoretically and practically. However, in this study group, the lack of awareness and adherence to MDG was reflected. This observation is similar to one of the local qualitative studies. This local study suggests that the possible explanation could be due to the lack of acknowledgment contributed by the government and non-governmental bodies in delivering to this special population [73].

On the other hand, for healthy eating, sugar is also frequently mentioned by parents, and parents categorize it as unhealthy food. There is still a lack of evidence claiming that sugar intake will cause children to change their behavior, such as hyperactivity. However, it does play a role in the general health and well-being of the child [74]. Last but not least, the argument between conventional food and organic food is also one of the concerns of the parent. Parents believe that conventional food will harm the development of their children as compared to organic food due to the risks related to pesticides, herbicides, and other chemicals used. Several scientific studies have been conducted to determine the nutritional value differences between conventional and organic foods. For instance, a study from Stanford University released a paper in 2012 stating that organic and conventional foods havethe same nutritional value [75]. Moreover, in 2014, another paper conducted by Europeans also came upon the same finding that both conventional and organic sources give the same level of nutrients [75]. Nevertheless, some studies were carried out to determine the health effect of organic and conventional food. Notably, positive associations were seen in the reduction rate of infertility, congenital disabilities, allergic, otitis media, pre-eclampsia, metabolic syndrome, high BMI, and non-Hodgkin lymphoma [76]. However, the evidence is still not strong to conclude how these differences impact the overall health benefits [77].

### 4.3. Parental Coping Strategies

The last topic explored in this qualitative study is the coping strategies of the parents. Notably, parents with special children will develop higher stress than typical development children [78,79,80]. The same authors that focus on mothers of children with autism spectrum disorder also reported that the high-stress level could be highly precipitated by social isolation, financial burden, and difficulty in getting medical treatment. All of these stressors added to a child’s behavior negatively impact the family. However, it is also noticed that, even with the high level of stressors, almost all families show resilient character and cope effectively [81]. Resilience is defined as a person’s ability to recover from trauma, negative experience, and hardships [82]. The findings occur with this study group. Parents of this study group reported a variety of coping strategies that are based on their experiences and also show resilience. In this study group, we theme parents’ coping strategies as problem or emotional-focused, with a few sub-themes such as support from family members, informational support, therapy services, religion, and grief and acceptance. To our knowledge, our findings are similar to a few studies [83,84,85].

Support from family members (spouse, extended family member, siblings)—according to studies, family, friends, or others that contribute to providing social support for the parents of children with special needs, are found to be the most determining factors in reducing the possible serious psychological and physiological effects [86]. In addition to that, another study also observed the positive outcomes on the parents’ quality of life when they receive support offered by family and friends [87]. Building a sound support system from different parties not only benefits the child with autism to cope with difficulties, but it also benefits the parents to improve their well-being and progressively develop resilience [85]. Getting support from the spouse is also frequently mentioned in this study. They feel that the spouse has the most important role. They see the necessity of sharing problems and solving issues together. They are dependent on each other for household responsibilities. Likewise, previous research explains this factor as one of the essential attributes of resilient autism families [81]. This also includes informal support from the same community, such as other parents of a child with an autism spectrum disorder [88].

Informational support—the parents in our study report the significance of equipping themselves with knowledge related to autism. Parents willingly join classes through social media, reading books, or learning from others. Parents think that this can help them build better confidence, better understand autism, and act as a self-preparing action if problems arise from their child. The majority of parents are more focused on developing their child’s basic skills, including eating. Similarly, a study conducted on Malaysian parents’ children with autism spectrum disorder also shared a similar finding of the importance of knowledge in helping them to cope and develop a better feeling of self-efficacy, and eventually strengthen them in general, which also helps in increasing their resilience [85]. This is also supported by another two studies [89,90] that agree on the importance of the knowledge that can indirectly help in one sense of competence, thereby allowing parents to control the situation.

Use of therapy services—to date, there is not yet any evidence-based intervention that has been shown to treat autism spectrum disorder; however, there are several therapy services that have come about to reduce the symptoms, improve cognitive function and fundamental skills, and enhance the child’s ability to function and get involved with the community [29,91,92,93,94,95]. The types of therapy services can be categorized into behavior and communication, dietary, medication, or complementary and alternative medicine [3]. Furthermore, therapy services are essential, especially for younger age children with autism, as this will help form a base for the child to practice the skill better as they grow. In Malaysia, children with special needs can receive therapy services from either a governmental organization (Community-Based Rehabilitation—PDK), a non-governmental organization (NASOM—National Autism Society of Malaysia), or a private-based practice occupational therapist center. The majority of parents will send their children to CBR. However, some parents will send their children to all these places to help their children improve faster. In this study group, the parents feel relief and lesser stress when sending their child for early intervention programs. This is supported by one study that this coping strategy can give parents a break while the child is attending classes. Parents can also, utilize this opportunity to receive consultation about the proper handling methods [96].

Religion—the parents in this study group hold a firm spiritual faith, indirectly increasing their confidence to cope better with their child. They think that God selects them for specific reasons. They also practice the teaching that everyone must be grateful and never compare one with others. This finding is somehow similar to other studies [85,88,97,98]. Simmons 2019 also found that faith was an essential factor depended on by some parents to provide the inner strength to cope well [99]. This can conclude that parents instilled the teachings of their religion to enable them to cope.

Grief and acceptance—this is reflected in our study group participants, which is also similar to previous findings [85,100,101,102,103,104]. In addition to that, specifically from early findings, grief has been reflected as a general response when knowing an autism diagnosis [105]. There are five stages of the grieving process, but an individual might not necessarily go through all these stages. Grief is a response to lose that everyone will experience [106]. On the other hand, the parents of this study group mentioned that once they accepted their child’s diagnosis, they could adapt better. The possible explanation is that as time passes, parents eventually learn and are aware that their thinking will reflect on the outcome. Henceforth, parents started to change their thinking and adapt accordingly. Adaptation is also known as the healing process for grieving parents and family members [85]. Parents of this study group reflect a solid inner strength to cope with the challenges of their autistic children. The current findings show that despite the challenges experienced by them, our parents’ group is able to cope with the available resources around them and cheer with their achievements. This, in turn, helps build resilience [85]. This finding also further supports previous studies’ suggestions that strengths and coping strategies are essential to parents’ psychosocial well-being, which can help reduce anger, anxiety, and depression among parents of children with autism [107].

## 5. Conclusions

This study highlights nutrition-related issues such as the eating behavior of children with ASD, the parents’ awareness about healthy eating, and the coping strategies used by parents to deliver a better nutrition outcome. This study group of children with ASD showed similar findings on eating behavior such as feeding problems, yet the parents were able to cope with the challenges and believed that nutrition is vital for their child’s growth and development. Parenting style, regardless of whether permissive, authoritative, authoritarian, or ignorant, should be further emphasized in future studies. Coping strategies help parents to accept and adapt to their child’s diagnosis. This helps parents be actively involved in their child’s growth milestones, and children with ASD can benefit greatly by including parents or caretakers in intervention programs [108].

## Figures and Tables

**Table 1:** Interview guide for the parents with children with ASD.

First, I will ask you some questions about your child’s eating behavior.
(1) Do you agree with the statement that children with ASD have a more problematic issue during eating time? Please tell me how it relates to your child’s eating behavior.
Second, I will ask related to healthy eating.
(1) What do you think about healthy eating? (2) Do you think it is essential to practice healthy eating for your child? If yes, why do you think so? Please tell me your experiences on how to help your child eat healthily.
Third, I will ask about coping strategies. Some studies showed that taking care of autistic children experiences higher stress levels than typical children.
(1) What do you think about this statement? Please share with me your experiences and your coping strategies.

**Table 2:** An overview of the thematic analysis process from initial quotes to themes.

Initial Quotes	Codes	Themes
“For my child, I noticed that he is quite selective with his food; he prefers to only to eat his favorite food, which consist of a small selection of assorted local *kuih (dessert)*, fruits, and colored rice. These are the main foods for him. So, for my part, I will only add on his few favorite side dishes such as anchovies and or egg.” (P3) “...my child refuses to eat. She only eats vermicelli noodle soup. She will keep throwing tantrums, screaming and shouting…as if we are torturing her.” (P5)	Limited food repertoire up to refusing to eat.	Feeding problems
“[Laughter from a parent] My child likes spicy food. He refuses any food that comes with clear-soup based. Something like sambal (*chili*) chicken or curry fish, he will finish the food. If the foods color combination looks dull, he refuses to eat too.” (P4)	Being very selective with the food presentation, only to one’s liking.	
“During his eating time, my child will fully focus while eating. We are not allowed to disturb him. And if he already selects an eating place, he will not go to other places. And even for the eating utensils, he also got his specific plate colors.” (P9)	Selective in designated place and utensils	

**Table 3:** Demographics.

	N (%)
Child	
Male	11 (73%)
Female	4 (27%)
Child age (years old)	
5	3 (20%)
6	2 (13%)
7	4 (27%)
8	1 (7%)
9	1 (7%)
10	2 (13%)
11	2 (13%)
Parent education level	
Completed degree and above	10 (67%)
Primary and secondary	5 (33%)
Parent occupations	
Housewife	7 (47%)
Public officers	5 (33%)
Self-employed	3 (20%)
Total household income	
Less than RM 4339	15 (100%)

## Data Availability

The dataset analyzed in this study is available on request through the correspondence author.
